# COVID-19 Vaccination Acceptance Among Healthcare Workers and Non-healthcare Workers in China: A Survey

**DOI:** 10.3389/fpubh.2021.709056

**Published:** 2021-08-02

**Authors:** Ming-Wei Wang, Wen Wen, Nan Wang, Meng-Yun Zhou, Chun-yi Wang, Jie Ni, Jing-jie Jiang, Xing-wei Zhang, Zhan-Hui Feng, Yong-Ran Cheng

**Affiliations:** ^1^Metabolic Disease Center, Affiliated Hospital of Hangzhou Normal University, Hangzhou, China; ^2^Department of Anesthesiology, The Second Hospital of Anhui Medical Unviersity, Hefei, China; ^3^Department of Molecular & Cellular Physiology, Shinshu University School of Medicine, Matsumoto, Japan; ^4^Neurological Department, Affiliated Hospital of Guizhou Medical University, Guiyang, China; ^5^School of Public Health, Hangzhou Medical College, Hangzhou, China

**Keywords:** COVID-19 vaccine, healthcare workers, non-healthcare workers, vaccine hesitant, vaccine resistant, vaccine acceptance

## Abstract

**Background:** The coronavirus pneumonia is still spreading around the world. Much progress has been made in vaccine development, and vaccination will become an inevitable trend in the fight against this pandemic. However, the public acceptance of COVID-19 vaccination still remains uncertain.

**Methods:** An anonymous questionnaire was used in Wen Juan Xing survey platform. All the respondents were divided into healthcare workers and non-healthcare workers. Multinomial logistic regression analyses were performed to identify the key sociodemographic, cognitive, and attitude associations among the samples of healthcare workers and non-healthcare workers.

**Results:** A total of 2,580 respondents completed the questionnaire, including 1,329 healthcare workers and 1,251 non-healthcare workers. This study showed that 76.98% of healthcare workers accepted the COVID-19 vaccine, 18.28% workers were hesitant, and 4.74% workers were resistant. Among the non-healthcare workers, 56.19% workers received the COVID-19 vaccine, 37.57% workers were hesitant, and 6.24% workers were resistant. Among the healthcare workers, compared with vaccine recipients, vaccine-hesitant individuals were more likely to be female (AOR = 1.52, 95% CI: 1.12–2.07); vaccine-resistant individuals were more likely to live in the suburbs (AOR = 2.81, 95% CI: 1.44–3.99) with an income of 10,000 RMB or greater (AOR = 2.00, 95% CI: 1.03–3.90). Among the non-healthcare workers, vaccine-hesitant individuals were more likely to be female (AOR = 1.66, 95% CI: 1.31–2.11); vaccine-resistant individuals were also more likely to be female (AOR = 1.87, 95% CI: 1.16–3.02) and older than 65 years (AOR = 4.96, 95% CI: 1.40–7.62). There are great differences between healthcare workers and non-healthcare workers in their cognition and attitude toward vaccines.

**Conclusions:** Our study shows that healthcare workers are more willing to be vaccinated than non-healthcare workers. Current vaccine safety issues continue to be a major factor affecting public acceptance, and to expand vaccine coverage in response to the COVID-19 pandemic, appropriate vaccination strategies and immunization programs are essential, especially for non-healthcare workers.

## Background

In the absence of a vaccine or effective treatment, all the countries around the world are trying to control the spread of COVID-19 by imposing quarantines and lockdowns, social distance measures, use of face masks in communities at all times, and travel restrictions. All these actions have caused enormous damage to people's physical and mental health and contributed to a significant global economic downturn (1–3). Therefore, there is a great need for an effective vaccine to control COVID-19. In fact, COVID-19 vaccines are being developed. According to the WHO, as of 12 March 2021, 81 vaccine candidates have been submitted for clinical evaluation, and 182 vaccine candidates have been submitted for preclinical evaluation (4, 5).

However, many side effects of vaccine have been reported in clinical evaluation, such as injection site pain (89.8%), fatigue (62.2%), headache (45.6%), and muscle pain (37.1%) (6). More serious side effects have also been reported, such as COVID-19 vaccine-associated immune thrombosis and thrombocytopenia (7). Despite great progress in vaccine development, there are still significant challenges in future immunization against COVID-19, one of which is uncertainty about the public acceptance of COVID-19 vaccination (8). A representative data from the general adult populations of Ireland and the United Kingdom showed that vaccine hesitancy/resistance was evident for 35 and 31% of these populations, respectively (9).

In fact, vaccine acceptance reflects the general perception of disease risk, vaccine attitudes, and needs of the general population, which is critical to the success of immunization programs to achieve a high vaccination coverage, especially for emerging infectious diseases (10, 11). Mathematical models showed that if the COVID-19 vaccine is 80% effective, then the coverage must be at least 75% to eliminate an ongoing pandemic (12). Therefore, it is extremely important to keep track of the public's views on vaccination, especially the views and acceptance of the vaccine among healthcare workers and non-healthcare workers.

Healthcare workers are often on the front lines of fight against epidemics, some of whom are required to carry out procedures with a high risk of contracting pathogens (13). In addition, previous studies reported that clinicians are an important source of vaccine information, and communication among clinicians can improve the adherence to vaccination recommendations (14–17). Certainly, healthcare workers are also concerned about the side effects of vaccines. Therefore, we need to know whether there is a difference in the acceptance of vaccines between the healthcare workers and non-healthcare workers in China. What factors affect the people's acceptance of the vaccine? This information is critical to prepare well for future vaccination strategies and immunization programs against COVID-19. Therefore, our study aimed to evaluate the acceptance of future COVID-19 vaccination, preference for vaccine attributes and schedules, and influencing factors for vaccination acceptance among healthcare workers and non-healthcare workers in China.

## Methods

### Population and Sampling

This study is a nation-wide cross-sectional study in China; the ethics committee of Affiliated Hospital of Hangzhou Normal University approved all the procedures performed. In January 2021, an anonymous online cross-sectional survey was conducted on Wen Juan network (https://www.wenjuan.com), founded by Shanghai Zhongyan Network Technology. It is the largest free online survey platform in China, which can provide questionnaire creation, release, management, collection, and analysis services for enterprises or individuals. Their personal information can be confirmed, and authentic, diverse, and representative samples can be obtained. The target population of this study is Chinese adults living in China, divided into healthcare workers and non-healthcare workers according to their occupation.

First, we calculated the sample size of the survey, according to a previous study, currently the average proportion of people accepted to be vaccinated is 64.9% (8). Therefore, an approximate value of 65% was selected for the calculation of sample size. The expected error rate was set to 1.5%. The sample size was calculated as follows (18, 19):

$$\begin{array}{l} \text{n}=\frac{u_{a}p\left(1-p\right)}{\delta^{2}} \end{array}$$

where *u*_*a*_ = 1.96, *p* is the proportion of vaccinations accepted, and δ is the standard deviation (0.015). To reduce the sampling error and increase the study power, a rough estimation was made by multiplying the calculated sample size by 1.35 times, leading to a final sample size of 2,675. The validation of the questionnaire is 0.83. After sorting out the collected questionnaires, we excluded the invalid questionnaires (lack of information, filling errors) and finally included 2,580 valid questionnaires. Among them, 1,329 questionnaires were from the healthcare workers, and 1,251 questionnaires were from the non-healthcare workers.

### Questionnaire Design

Informed consent has been designed. In the first part of the survey page, we have set that the respondents can continue to complete the questionnaire if they agree to response to it. If they do not agree, they can not go on. A self-administered questionnaire was designed based on previous study to evaluate the acceptance of vaccines for emerging infectious diseases (20). The questionnaire mainly includes two parts: social demographic characteristics and cognition and attitude toward vaccine. The first part focused on “gender, age, education level, living area, family income, and health status.” The second part featured eight questions about cognition and attitude toward vaccine. The scenario presented to the physicians was as follows: “Do you know about COVID-19 vaccine development and vaccination; do you think the COVID-19 vaccine has side effects; do you think it's better to get immunity to infectious diseases naturally than vaccinating; do you think vaccination is an effective way to prevent and control the epidemic; do you believe in the safety and effectiveness of vaccines; do you know the vaccination place in your residential area; does your work unit encourage you to get vaccinated; do you think vaccination has an effect on regional epidemic prevention.” Attitude toward vaccine including three types: resistance, hesitation, and acceptance. Resistance means rejection of vaccination. Hesitation means unwillingness to accept because of being uncertain, worried, or embarrassed about vaccination. Acceptance means agreeing with an inoculation. The three types of attitudes toward vaccines were investigated through questions: would you like to get the COVID-19 vaccine? The answer option set three options: resistance, hesitation, and acceptance.

### Statistical Method

A descriptive statistical analysis was conducted on the social and demographic characteristics of the sample, and the corresponding proportion was calculated. Multiple logistic regression analysis was used, in which the dependent variable was the vaccine acceptance (resistance, hesitation, and acceptance), and the independent variable was the influencing factor (social population, cognition, and attitude). For these analyses, the vaccine acceptance group was set as the reference category to identity factors associated with vaccine hesitancy and vaccine resistance. All relationships between the predictor and criterion variables are represented as adjusted odds ratios (AOR) with 95% confidence intervals. The statistical tests were two-sided, and the effects with *p* < 0.05 were considered to be statistically significant. All statistical models were constructed using R software version 3.6.0 (R Foundation for Statistical Computing, version 3.6.1; http://www.Rproject.org).

## Results

A total of 2,580 respondents completed the questionnaire, including 1,329 healthcare workers and 1,251 non-healthcare workers (Table 1). All of them were distributed in 33 provincial administrative regions in China (Supplementary Table 1).

**Table 1:** Sociodemographic characteristics of healthcare workers and non-healthcare samples.

	**Health care (*N* = 1,329)%**	**Non-health care (*N* = 1,251)%**
**Gender**		
Male	471 (35.4)	603 (48.2)
Female	858 (64.6)	548 (51.8)
**Age**		
18–24	157 (11.8)	363 (29.0)
25–34	535 (40.3)	278 (22.2)
35–44	414 (31.2)	354 (28.3)
45–54	182 (13.7)	153 (12.3)
55–64	34 (2.5)	87 (6.5)
≥65	7 (0.5)	16 (1.7)
**Education**		
Senior high school and below	18 (1.4)	235 (18.8)
Vocational college or junior college	221 (16.6)	420 (33.6)
Undergraduate	820 (61.7)	540 (43.2)
Graduate student	270 (20.3)	156 (4.4)
**Residential area**		
Countryside	111 (8.3)	37 (3.0)
Suburb	42 (3.2)	216 (17.3)
City	1176 (88.5)	998 (79.7)
**Income(RMB)**		
≤ 5,000	480 (36.1)	614 (49.1)
5,000–10,000	264 (19.8)	262 (20.9)
≥10,000	585 (44.1)	375 (30.0)
**Chronic disease**		
Yes	120 (9.1)	133 (10.6)
No	1209 (90.9)	1118 (89.4)
**Is there an infected COVID-19 around**		
Yes	11 (0.8)	5 (0.4)
No	1318 (99.2)	1246 (99.6)

**Figure 1:**
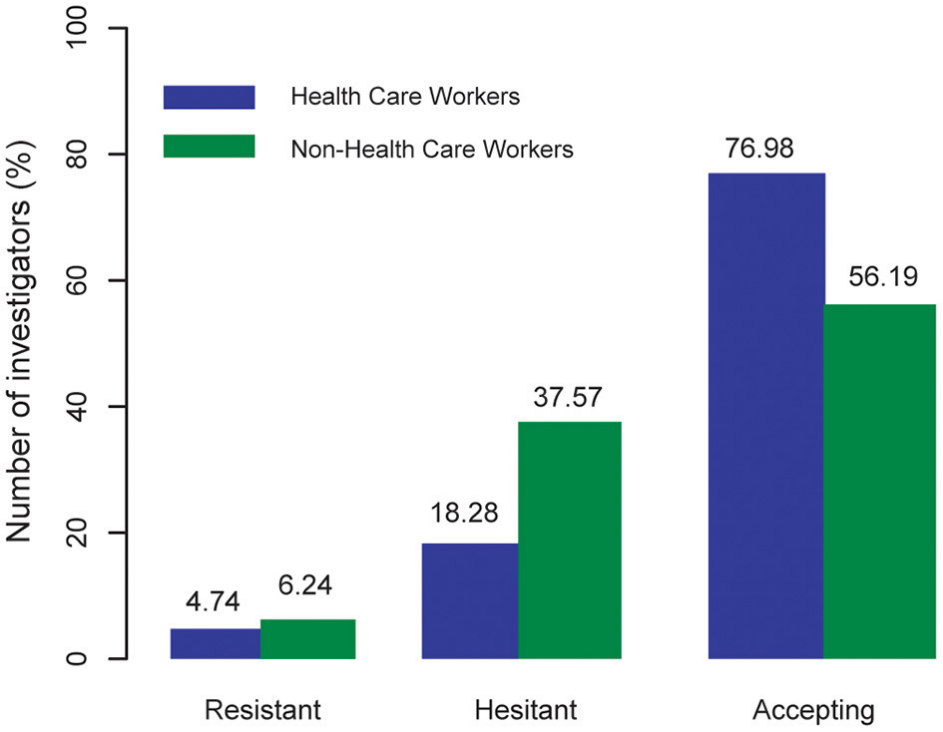
Rates of COVID-19 vaccine acceptance, hesitance, and resistance.

The basic characteristics of the respondents showed that 76.98% of healthcare workers accepted the COVID-19 vaccine; 18.28% of healthcare workers were hesitant; and 4.74% of healthcare workers were resistant. Among the non-healthcare workers, 56.19% of non-healthcare workers received the COVID-19 vaccine, significantly lower than that of healthcare workers; 37.57% non-healthcare workers were hesitant; and 6.24% non-healthcare workers were resistant, higher than that of healthcare workers (Figure 1).

The correlations of sociodemographic and health indicators among the samples of healthcare workers and non-healthcare workers showed that vaccine-hesitant individuals were more likely to be female (AOR = 1.52, 95% CI: 1.12–2.07) among the healthcare workers, comparing with vaccine recipients. Vaccine-resistant individuals were more likely to live in the suburbs (AOR = 2.81, 95% CI: 1.44–3.99) with an income of 10,000 RMB or greater (AOR = 2.00, 95% CI: 1.03–3.90). Among the non-healthcare workers, vaccine-hesitant individuals were more likely to be female (AOR = 1.66, 95% CI: 1.31–2.11). Vaccine-resistant individuals were also more likely to be female (AOR = 1.87, 95% CI: 1.16–3.02) and older than 65 years (AOR = 4.96, 95% CI: 1.40–7.62) (Table 2).

**Table 2:** Relationships of sociodemographic and health indicators among the samples of healthcare workers and non-healthcare workers.

	**Health care workers**				**Non-health care workers**			
	**Vaccine hesitant**		**Vaccine resistant**		**Vaccine hesitant**		**Vaccine resistant**	
	**AOR**	**95%CI**	**AOR**	**95%CI**	**AOR**	**95%CI**	**AOR**	**95%CI**
**Gender**								
Male	Ref							
Female	1.52	**1.12–2.07**	1.03	0.61–1.74	1.66	**1.31**–**2.11**	1.87	**1.16**–**3.02**
**Age**								
18–24	Ref							
25–34	0.53	**0.35**–**0.80**	0.97	0.41–2.30	1.18	0.85–1.64	1.03	0.71–2.11
35–44	0.54	**0.35**–**0.80**	0.89	0.36–2.17	1.16	0.85–1.58	1.34	0.71–2.53
45–54	0.25	**0.13**–**0.46**	0.96	0.35–2.60	0.91	0.61–1.35	0.71	0.28–1.84
55–64	0.48	0.19–1.25	–	–	1.13	0.68–1.88	3.04	**1.38**–**6.72**
≥65	0.90	0.17–4.83	–	–	0.54	0.14–2.01	4.96	**1.40**–**7.62**
**Education**								
Senior high school and below	Ref							
Vocational college or junior college	0.78	0.26–2.30	0.60	0.07–5.17	0.62	**0.44**–**0.89**	0.26	**0.10**–**0.64**
Undergraduate	0.53	0.18–1.52	0.73	0.09–5.71	0.94	0.68–1.30	1.15	0.63–2.13
Graduate student	0.51	0.17–1.53	0.86	0.10–7.04	1.10	0.72–1.69	1.19	0.54–2.65
**Residential area**								
Countryside	Ref							
Suburb	0.46	0.16–1.29	2.81	**1.44**–**3.99**	1.15	0.56–2.36	1.58	0.31–8.00
City	0.68	0.43–1.07	1.14	0.70–1.62	0.88	0.65–1.21	1.81	0.85–3.86
**Income**								
≤ 5,000	Ref							
5,000–10,000	0.89	0.65–1.21	1.17	0.63–2.16	1.35	**1.03–1.77**	0.99	0.56–1.75
≥10,000	1.03	0.70–1.52	2.00	**1.03**–**3.90**	1.56	**1.15–2.12**	1.63	0.92–2.89
**Is there an infected COVID-19 around**								
No	Ref							
Yes	1.81	0.47–7.07	2.34	0.28–7.33	0.37	0.04–3.35	0.11	0.04–1.63
**Chronic disease**								
Yes	Ref							
No	1.38	0.87–2.18	1.61	0.74–3.48	0.89	0.60–1.31	1.67	0.88–3.18

*Reference, vaccine acceptance. AOR adjusted odds ratios, 95% CI: 95% confidence intervals for the adjusted odds ratios; statistically significant associations (p < 0.05) are highlighted in bold*.

The cognition and attitude of healthcare workers and non-healthcare workers are described in Table 3. The reasons for vaccine hesitation or resistance among healthcare workers are as follows: Adverse effects of vaccine were considered (AOR = 6.91, 95% CI: 4.73–10.1; 9.30, 95% CI: 4.20–20.6). Vaccines were not considered an effective method for controlling epidemics (AOR = 2.43, 95% CI: 1.52–3.88; 5.40, 95% CI: 2.85–10.2). The safety and efficacy of the vaccine were not convinced (AOR = 11.71, 95% CI: 6.78–20.23; 40.77, 95% CI: 22.0–83.1). Work unit had no vaccination requirements (AOR = 4.61, 95% CI: 3.32–6.41; 2.10, 95% CI: 1.04–3.89). In addition, people with vaccine resistance were more likely to perceive vaccination as having no significant or moderate impact on district prevention (AOR = 21.6, 95% CI: 11.6–27.5; 11.93, 95% CI: 8.0–17.2).

**Table 3:** Relationships of cognitive and attitude with the samples of healthcare workers and non-healthcare workers.

	**Health care workers**				**Non-health care workers**			
	**Vaccine hesitant**		**Vaccine resistant**		**Vaccine hesitant**		**Vaccine resistant**	
	**AOR**	**95%CI**	**AOR**	**95%CI**	**AOR**	**95%CI**	**AOR**	**95%CI**
**Do you know about COVID-19 vaccine development and vaccination**								
Yes	Ref							
No	1.53	0.73–3.18	2.41	0.82–7.09	1.42	0.94–2.15	2.32	**1.18**–**4.58**
**Do you think the COVID-19 vaccine has side effects**								
No	Ref							
Yes	6.91	**4.73**–**10.1**	9.30	**4.20**–**20.6**	3.96	**3.01**–**5.22**	4.50	**2.44**–**8.31**
**Do you think it's better to get immunity to infectious diseases naturally than vaccinating**								
Yes	Ref							
No	0.60	**0.41**–**0.89**	0.23	**0.13**–**0.39**	0.58	**0.42**–**0.81**	0.36	**0.21**–**0.68**
**Do you think vaccination is an effective way to prevent and control the epidemic**								
Yes	Ref							
No	2.43	**1.52**–**3.88**	5.40	**2.85**–**10.2**	2.58	**1.73**–**3.75**	5.35	**3.04**–**9.40**
**Do you believe in the safety and effectiveness of vaccines**								
Yes	Ref							
No	11.71	**6.78**–**20.23**	40.77	**22.0**–**83.1**	9.81	**6.04**–**15.9**	35.99	**19.3**–**66.9**
**Do you know the vaccination place in your residential area**								
Yes	Ref							
No	1.06	0.76–1.47	0.71	0.37–1.39	1.08	0.85–1.36	0.84	0.52–1.34
**Does your work unit encourage you to get vaccinated**								
Encouraged	Ref							
Discouragement	2.92	0.26–4.41	9.11	0.81–10.3	1.72	0.42–7.02	9.00	**2.08**–**11.9**
No request	4.61	**3.32**–**6.41**	2.10	**1.04**–**3.89**	3.45	**2.63**–**4.52**	2.75	**1.59**–**4.77**
**Do you think vaccination has an effect on regional epidemic prevention**								
Important	Ref							
Commonly	18.9	9.28–38.61	11.93	**8.0**–**17.2**	5.43	**3.51**–**8.42**	13.08	**7.12**–**24.02**
Unimportance	4.98	0.70–35.56	21.6	**11.6**–**27.5**	1.18	**0.20**–**7.10**	19.1	**7.07**–**23.4**

*Reference = vaccine acceptance. AOR adjusted odds ratios, 95% CI: 95% confidence intervals for the adjusted odds ratios; statistically significant associations (p < 0.05) are highlighted in bold*.

Among the non-healthcare workers, those with vaccine hesitation were more likely to believe that vaccination had an average impact on regional prevention (AOR = 5.43, 95% CI: 3.51–8.42), except for the above reasons which were similar to those of healthcare workers. Non-healthcare workers with vaccine resistance were more likely to be unaware of vaccine development and vaccination (AOR = 2.32, 95% CI: 1.18–4.58) and discouraged by their employer from getting vaccinated (AOR = 9.00, 95% CI: 2.08–11.9).

## Discussion

Our results showed that the acceptance rate of vaccine was higher than 50% in both healthcare workers and non-healthcare workers. Although our study showed a high acceptance of vaccination among the healthcare workers, not all of them wanted to receive the vaccine. The acceptance rate of healthcare workers was significantly higher than that of non-healthcare workers (76.98 vs. 56.19%), while the rates of vaccine hesitation and resistance were lower than those of non-healthcare workers. We think they also have an attitude toward the vaccine and what concerns they might have although they had been vaccinated. Their experience might provide better suggestions for our vaccination strategy. Therefore, we did not exclude this part of the vaccinated population before the investigation.

Females were more likely to show signs of vaccine hesitancy and resistance than males, especially among non-healthcare workers. In terms of age, vaccine resistance among the non-healthcare workers was more common in people over 55 years old. In addition, we found that the education level and the presence of chronic diseases and COVID-19 infection had no significant influence on the acceptance of vaccine among the healthcare workers, while the residence and income level had some influence. In contrast, among the non-healthcare workers, education level had a certain effect on vaccine resistance and resistance. Therefore, to increase the vaccine coverage and eliminate the ongoing pandemic, non-healthcare workers should be encouraged to actively vaccinate themselves, while the acceptance of healthcare workers to vaccinate should not be ignored.

Previous reports indicated that the acceptance to get vaccinated lies between 60 and 90% among the doctors in Greece (February 2020) and France (March–July 2020), and between 40 and 60% among the nurses in Hong Kong, China (February–March 2020) (21–23). Numerous studies had reported that clinicians were an important source of vaccine information. Communication among physicians could improve the adherence to vaccination recommendations (24, 25), and vaccinated healthcare workers were more likely to recommend vaccines to friends, families, and their patients (26–29). Therefore, it is necessary to evaluate the acceptance of vaccination by healthcare workers and its influencing factors, and to motivate the non-healthcare workers to actively respond to vaccination through them.

Concerns about the safety or side effects of the vaccine were reported to be the main reason for the hesitation, and previous study on the acceptance of vaccination against emerging serious infectious diseases such as H1N1 had also emphasized that uncertainty about the new vaccine, particularly its safety, can reduce the confidence in the vaccine and thus the acceptance (30). Our findings were consistent with these findings. The common factors influencing vaccine hesitation or resistance among healthcare workers and non-healthcare workers were as follows: (1) They thought that the vaccine had side effects. (2) They did not think that vaccination was an effective way to prevent and control the epidemic. (3) They did not believe in the safety and effectiveness of the vaccine (the main reason). (4) The current cost of vaccines was unacceptable. (5) There was no requirement for vaccination in the work unit. We found that the reasons for vaccine hesitation and resistance among the non-healthcare workers were not only related to the above points, but also related to their lack of knowledge about the development and vaccination of COVID-19 vaccine.

We also found that the non-healthcare workers were less concerned about the side effects and safety of vaccines than the medical workers, probably because of the lack of medical knowledge and low understanding of COVID-19 vaccines. Universal vaccination is an important measure to control the epidemic. To control COVID-19 effectively and quickly and restore social activities, appropriate vaccination strategies, and immunization programs should be designed to increase the coverage, especially among those who are hesitant about vaccination (16). Currently, the cost of the vaccine should be affordable to the public, and China has long stated its goal is to make its COVID-19 vaccine a global good when it is ready for use (6, 31). In response to public concerns about the safety and efficacy of vaccines, health professionals, and credible authorities, such as governments or other sources, should actively organize health education and communication to combat disinformation and misinformation, and disseminate authoritative information in a transparent manner, especially about vaccine effectiveness, and adverse events (32–36). This will help to encourage community leaders of healthcare professionals and surrounding friends or relatives to share their personal experiences with COVID-19 vaccination to build vaccine confidence and trust (37). In addition, a high perception of benefits and low perceived barriers to receiving the vaccine were the two most important factors influencing a definite intention for COVID-19 vaccination; hence, public health intervention programs should focus on increasing the perception of benefits of vaccination (1).

This is the first study on a large-scale vaccination of healthcare workers and non-healthcare workers during the COVID-19 pandemic. This study provides guidance for the vaccination of Chinese population, especially for non-healthcare workers.

This study has some limitations. First, in this study, an online questionnaire was used, and the public may have problems such as information deviation or false filling when filling in the questionnaire, which requires further research. Second, we used a web push technology to make the survey. We could not obtain a balanced feedback from different cities in China. From S-table, the regional imbalance of responders was obvious. However, we have to point out that the number of medical staff and non-medical staff was relatively balanced.

## Conclusions

Chinese adults have a high degree of acceptance of vaccination, and healthcare workers are more willing to be vaccinated than non-healthcare workers. Current vaccine safety issues are a major factor affecting public acceptance, and to expand vaccine coverage in response to the COVID-19 pandemic, appropriate vaccination strategies and immunization programs are essential.

## Data Availability Statement

The original contributions presented in the study are included in the article/Supplementary Material, further inquiries can be directed to the corresponding author/s.

## Ethics Statement

This study is a nation-wide cross-sectional study in China; the ethics committee of Affiliated Hospital of Hangzhou Normal University approved all the procedures performed.

## Author Contributions

Y-RC and Z-HF conceived the study and designed the analysis. Y-RC and NW performed statistical analysis. M-WW and WW wrote the first draft of the manuscript. M-YZ, JN, J-jJ, C-yW, and X-wZ participate in revision the manuscript. All authors contributed to revision of the manuscript.

## Conflict of Interest

The authors declare that the research was conducted in the absence of any commercial or financial relationships that could be construed as a potential conflict of interest.

## Publisher's Note

All claims expressed in this article are solely those of the authors and do not necessarily represent those of their affiliated organizations, or those of the publisher, the editors and the reviewers. Any product that may be evaluated in this article, or claim that may be made by its manufacturer, is not guaranteed or endorsed by the publisher.
