# Supplementary material for: COVID-19 Vaccination Acceptance Among Healthcare Workers and Non-healthcare Workers in China: A Survey
Source: Front Public Health. 2021 Aug 2;9:709056. doi: 10.3389/fpubh.2021.709056 (PMC8364953; doi:10.3389/fpubh.2021.709056)
Supplement: Supplementary file 1 [file Table_1.docx]

**S-Table 1. Provincial distribution of respondents (*N* = 2,580) of the survey on COVID-19 vaccination in China**

| Region | Number | percentage (%) |
| --- | --- | --- |
| Anhui | 267 | 10.35 |
| Beijing | 150 | 5.81 |
| Fujian | 46 | 1.78 |
| Gansu | 23 | 0.89 |
| Guangdong | 82 | 3.18 |
| Guangxi | 36 | 1.40 |
| Guizhou | 156 | 6.05 |
| Hainan | 32 | 1.24 |
| Hebei | 112 | 4.34 |
| Henan | 58 | 2.25 |
| Heilongjiang | 35 | 1.36 |
| Hubei | 78 | 3.02 |
| Hunan | 38 | 1.47 |
| Jiangsu | 127 | 4.92 |
| Jiangxi | 47 | 1.82 |
| Liaoning | 53 | 2.05 |
| Neimenggu | 27 | 1.05 |
| Ningxia | 37 | 1.43 |
| Qinghai | 31 | 1.20 |
| Shandong | 89 | 3.45 |
| Shanxi | 60 | 2.33 |
| Shannxi | 36 | 1.40 |
| Shanghai | 171 | 6.63 |
| Sichuan | 92 | 3.57 |
| Taiwan | 15 | 0.58 |
| Tianjin | 108 | 4.19 |
| Hong Kong | 16 | 0.62 |
| Xinjiang | 21 | 0.81 |
| Yunnan | 56 | 2.17 |
| Zhejiang | 357 | 13.84 |
| Chongqing | 89 | 3.45 |
| Xizang | 19 | 0.74 |
| Macao | 16 | 0.62 |
| Total | 2580 | 100 |
